# Repeat Uterine Artery Embolization for Obstetric Hemorrhages: A Rare Event in a Single Patient

**DOI:** 10.7759/cureus.29729

**Published:** 2022-09-29

**Authors:** Priyanjali Sinha, Neema Acharya, Pankaj Banode

**Affiliations:** 1 Obstetrics and Gynaecology, Datta Meghe Institute of Medical Sciences, Wardha, IND; 2 Obstetrics and Gynaecology, Datta Meghe Institute of Medical Science, Wardha, IND; 3 Interventional Radiology, Datta Meghe Institute of Medical Science, Wardha, IND

**Keywords:** interventional radiology, uterine artery embolization, mtp, hemorrhage, puerperium

## Abstract

This is a case report of a single patient who had two episodes of obstetric hemorrhages, first in the puerperium and second one post medical termination of pregnancy for which the patient had to be managed by uterine artery embolization (UAE). This is a rare case in which this interventional radiological procedure proved life-saving twice in her obstetric history.

A 29-year-old woman with an obstetric history of para two and living two (P2L2), presented with a complaint of per-vaginum (p/v) spotting with a history of recent intake of pills for medical termination of pregnancy (MTP). Retained products of conception (RPOC) were ruled out radiologically and she was managed conservatively. She subsequently presented with acute uterine bleed with severe anemia and lethargy. After initial resuscitation and failing of conservative therapy, she was promptly taken up for bilateral UAE. She had also undergone UAE 5 years ago during her puerperal period.

Uterine artery embolization is a safe and effective life-saving procedure to control torrential uterine bleed and should be considered when the conservative approach has failed.

## Introduction

Uterine artery embolization (UAE) has been increasingly accepted as a safe and effective method for hemostasis in obstetric hemorrhage which preserves menstruation as well as fertility [1]. UAE is one of the interventions that has shown promising results in the management of acute obstetrical hemorrhage [2].

UAE provides as a minimally invasive option for controlling postpartum hemorrhage (PPH) that is resistant to medical intervention [3]. Numerous studies have shown the reliability and effectiveness of UAE as a fertility-saving intervention for PPH [4]. However, problems following UAE, like uterine infection and ovarian malfunction, are thought to impact a woman's ability to conceive in the future and her ability to bear a child [5].

UAE is commonly indicated for uncontrolled uterine bleeding in cases of large fibroids in women with significant risk factors for surgery or women who wish to retain their uterus [6]. Prior to dilatation and curettage or hysteroscopic resection, it is occasionally done to reduce the risk of major hemorrhage caused by retained fetal products. It also serves as a life-saving procedure in postpartum and also in postabortion conditions [5].

UAE has turned out to be a minimally invasive technique with reduced morbidity and early recovery when compared with surgical options. However, there are a few absolute contraindications to this procedure which include suspected pregnancy, recent or ongoing pelvic sepsis, and severe allergy to radiographic contrast [6].

## Case presentation

A woman who had obstetric hemorrhage twice, the first one in a puerperium and the second one after medical termination of pregnancy in which there was an abnormal vascular pattern seen in the myometrial decidua, presented with acute hemorrhage in both the instances in which she underwent uterine artery embolization as the minimally invasive approach for obstetric hemorrhages.

A 29-year-old para two and living two (P2L2) presented to the hospital outpatient department (OPD) with pain in the lower abdomen associated with spotting in the last 6-7 days. There was a history of ingestion of MTP (medical termination of pregnancy) pills. She complained of increased menstrual flow for 3 days which was associated with the passage of clots.

Her routine blood investigations were normal except for mild anemia of 9 gm/dl. Her ultrasound scan showed a bulky uterus (endometrial thickness - 13 mm) with a retained product of conception, for which she was advised suction and evacuation but she defaulted and later presented to OPD after 10 days with a complaint of continued passage of clots per-vaginum (p/v) without any new symptoms. Her repeat USG (ultrasonography) scan showed no retained product of conception. There were no clinical and radiological findings suggestive of adenomyosis. She was advised medications and asked to revisit after 5 days. The patient then presented the following day with severe bleeding p/v. On examination, she was pale and dehydrated with tachycardia of 110 beats per minute. Her hemoglobin level was 6 gm/dl which was a significant drop of 3 gm/dl from the previous level. She was admitted and resuscitated with two units of packed red cells and intravenous crystalloids. She subsequently underwent uterine artery embolization as a life-saving procedure.

**Figure 1:**
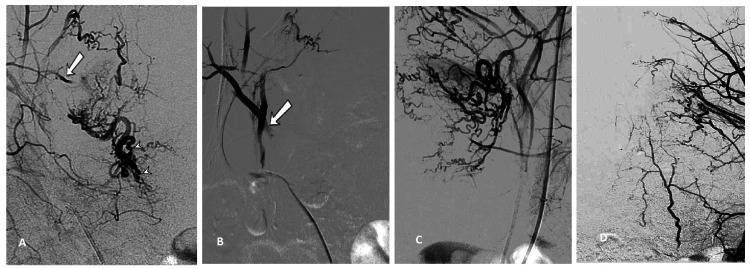
Angiographic image of embolization procedure A: tortuous left uterine artery (arrow) and uterine artery terminal branches (arrowheads); B: after embolization, the left uterine artery displays blood flow stasis (arrow); C and D: pre- and post-embolization of the right side.

The embolization procedure was carried out under conscious sedation. The uterine arteries were accessed through the femoral artery using a super-selective technique and a coaxial 2-3 French catheter was placed in each uterine artery in turn with the help of fluoroscopic guidance. Care was taken to avoid targeting non-uterine branches of the internal iliac artery. Each uterine artery was injected with 300-500 micrometers PVA (polyvinyl alcohol) particles until there was complete or near complete arterial stasis. It was essential to embolize both uterine arteries as cross-flow collateralization would have prevented adequate hemostasis. Intraprocedural angiography reveals a tortuous left uterine artery (Figure 1A arrow) and uterine artery terminal branches (marked by arrowheads), but there was no arteriovenous malformation.

Figure 1B is an angiographic image post embolization where the left uterine artery shows stasis of blood flow (arrow) and there was no active contrast extravasation. Similarly, Figure 1C and Figure 1D are pre- and post-embolization of the right side. Her post-procedural recovery was uneventful and the bleeding settled and the patient was discharged on combined oral contraceptives.

She had a similar event 5 years back, during her puerperal period, one month after a caesarean section when she presented with acute puerperal hemorrhage for which she underwent UAE as a life-saving procedure. This was a rare case where the same patient underwent UAE as an emergency life-saving procedure twice in her obstetric history.

## Discussion

The main factor contributing to maternal morbidity and mortality is still obstetric hemorrhage [7]. When there is unexpected, heavy, intermittent bleeding, especially after childbirth or uterine surgery, uterine arteriovenous malformation (AVM) is typically diagnosed.

UAE is a quick procedure that often takes under an hour to finish. A coaxial 2-3 French catheter is put in each uterine artery in turn after the uterine arteries are accessible through the femoral artery using a highly selective method using fluoroscopy. This small-sized coaxial catheter is strongly recommended to avoid arterial spasms. Each uterine artery receives injections of 300-500 micrometers PVA (poly-vinyl-alcohol) particles until there is complete or almost complete arterial stasis [6].

Both uterine arteries must be embolized because if one is left patent, cross-flow collateralization will cause an incomplete stasis of blood flow [8]. It's crucial to have a high index of suspicion for uterine AVM because intrauterine gynecologic examination methods like hysteroscopy or dilatation and curettage (D&C) may unintentionally worsen hemorrhage [2].

Aoki et al [9] did a retrospective study of 33 patients who underwent UAE for primary PPH and 85% had success while 15% had to go for a hysterectomy. They also identified as obvious arterial blood flow through the ovarian vessels as an important cause of failure of UAE and may need ovarian artery embolization along with UAE. In our case, both times the patient was successfully and effectively treated with UAE alone.

UAE is a safe, minimally invasive treatment for postpartum hemorrhage with a reported success rate of greater than 90% [10]. Additionally, this surgery may protect a woman's potential for future conception.

## Conclusions

Uterine artery embolization is a safe and effective life-saving procedure to control torrential uterine bleed and should be considered when a conservative approach to control uterine bleeding has failed. In this case, it has proved to be fertility-preserving and life-saving twice in the same patient, which prevented surgical exploration and associated morbidity.
